# Thyroid Metastasis From Caecal Adenocarcinoma

**DOI:** 10.7759/cureus.24120

**Published:** 2022-04-13

**Authors:** Sheikh Muktadir Bin Momin, Milord Hamal, Nicola Chaston, Eranga Nissanka-Jayasuriya, Ali Al-Lami

**Affiliations:** 1 Department of Otolaryngology, East Kent Hospitals University NHS Foundation Trust, Ashford, GBR; 2 Department of General Surgery, East Kent Hospitals University NHS Foundation Trust, Ashford, GBR; 3 Department of Pathology, East Kent Hospitals University NHS Foundation Trust, Ashford, GBR

**Keywords:** head and neck cancer surgery, thyroid cancer surgery, colorectal cancer, metastasis, thyroid cancer

## Abstract

The thyroid is a rare site of colorectal metastasis, comprising 0.1% of the surgical case series. A 62-year-old woman with caecal adenocarcinoma and previously surgically resected lung and liver metastases presented incidentally with a right thyroid nodule, which had grown and become symptomatic. Imaging revealed the nodule to have extracapsular spread, and cytology demonstrated metastatic adenocarcinoma. The patient underwent a technically challenging right thyroidectomy and neck dissection, with the final histopathological review demonstrating metastatic adenocarcinoma from a colorectal primary. The patient has subsequently undergone adjuvant radiotherapy. This case highlights an uncommon source of metastasis to the thyroid, which may aid clinicians to recognise and initiate treatment. It also highlights the technical challenges of performing surgery in such cases.

## Introduction

Colorectal cancer is the third-most-common cancer worldwide, accounting for 1.8 million new cases in 2018 [1]. 20% of cases present with metastases at diagnosis [2], with histological subtype and primary site factors influencing metastatic patterns. The liver, lung, and brain are common sites of metastasis [3] and are associated with poorer outcomes. Conversely, metastases are a rare cause of thyroid cancer, accounting for 1.4-3% of all patients undergoing surgery for suspected thyroid cancer. 48.1% of these are renal cells in origin, whilst 10.4% originate from colorectal cancer [4].

This paper presents a case of caecal adenocarcinoma metastasising to the thyroid gland several years after initial diagnosis, and we highlight the importance of identifying uncommon metastatic sites after previous curative primary treatment. We would also highlight the technical challenges in the surgical management of the case.

## Case presentation

A 62-year-old woman presented to the medical team in September 2015 with significant unexplained anaemia, requiring 3 units of blood transfusion. On further evaluation, she had right iliac fossa tenderness. She underwent colonoscopy and cross-sectional imaging, which revealed caecal cancer, and the patient underwent a laparoscopic right hemicolectomy on December 4, 2015, followed by adjuvant XELOX chemotherapy. She was on AJCC stage IIIc (T4bN1M0). On April 9, 2018, she underwent left lower lobectomy and video-assisted thoracoscopic surgery (VATS) for single lung metastasis, which was confirmed on histology to be a moderately differentiated adenocarcinoma consistent with a colorectal primary. On January 16, 2019, she underwent a partial right hepatectomy for solitary metastasis of segment 8 of the liver, and on August 17, 2020, she underwent a further right lower lobe resection and VATS for another right lung oligometastasis.

**Figure 1:**
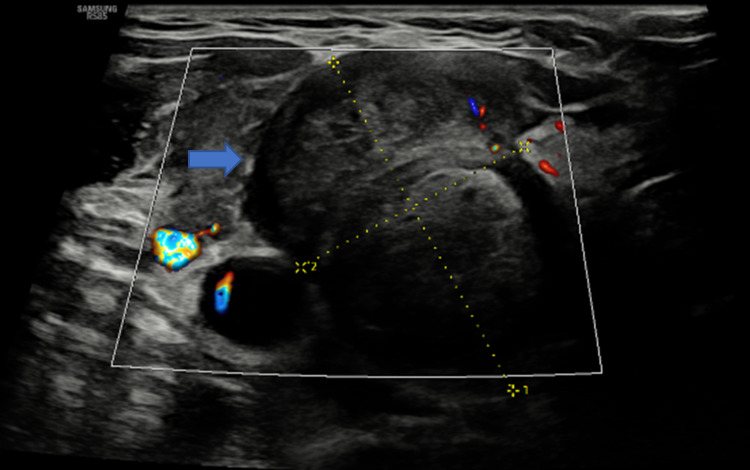
US thyroid This demonstrates a large solid hypoechoic nodule in the right thyroid lobe (blue arrow) with likely extra-capsular extension measuring 32 × 22 × 41 mm^3^ in dimension, graded a U5 thyroid nodule. An fine needle aspiration cytology (FNAC) was subsequently performed.

**Figure 2:**
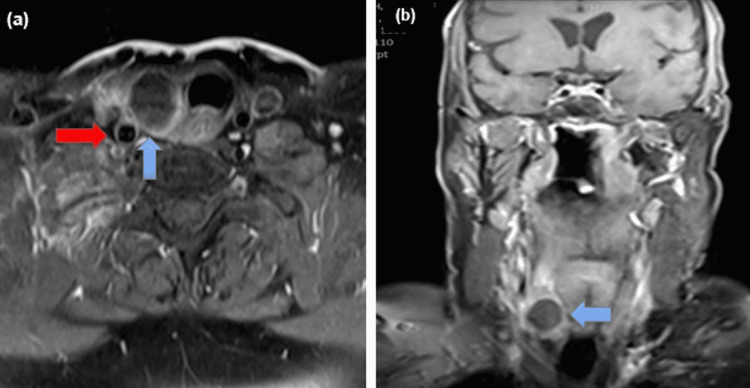
T1 MRI post-gadolinium Transverse (a) and coronal (b) sections demonstrating a right thyroid mass (blue arrow) with internal haemorrhage and laterally focal extra-capsular extension, closely applied to the right common carotid artery to a third of its circumference (red arrow).

**Figure 3:**
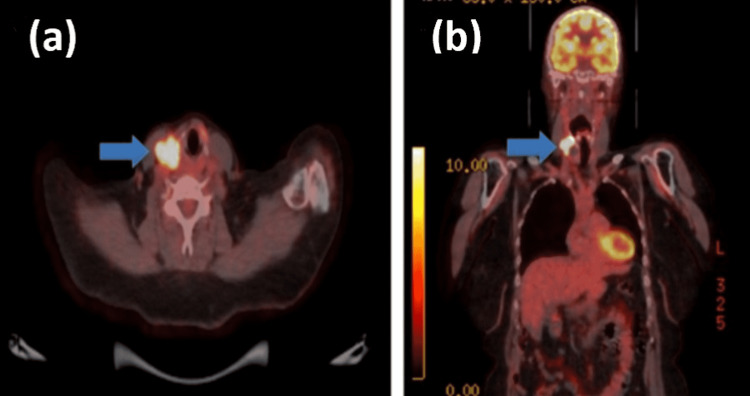
FDG-PET CT Transverse (a) and coronal (b) sections demonstrating a solitary FDG-avid lesion (blue arrow) in the right neck closely related to the right thyroid lobe, which was reported as a likely locally advanced malignancy rather than bowel cancer metastasis.

She remained under surveillance for colorectal cancer, with carcinoembryonic antigen (CEA) ranging between <1.0-and 1.3. During this period, she was found incidentally to have a right thyroid nodule and was referred to our Head and Neck service. She reported that the nodule had increased in size and had become more symptomatic. There was no dysphagia or dysphonia, and her thyroid function tests were normal. On examination, she had a palpable 2 cm right thyroid nodule. She underwent an ultrasound-guided fine-needle aspiration of the nodule (Figure 1), where cytology revealed a differential diagnosis of poorly differentiated thyroid carcinoma or metastasis (Thy5). She also underwent an MRI neck and (18)F-fluorodeoxyglucose positron emission tomography (FDG-PET) of the whole body (Figures 2-3), and after multidisciplinary team discussion, was listed for a right hemithyroidectomy.

Given the unusual site (thyroid) and time gap after initial colorectal cancer diagnosis (six years), differentials alongside metastatic thyroid carcinoma included primary thyroid malignancies, particularly medullary thyroid cancer.

**Figure 4:**
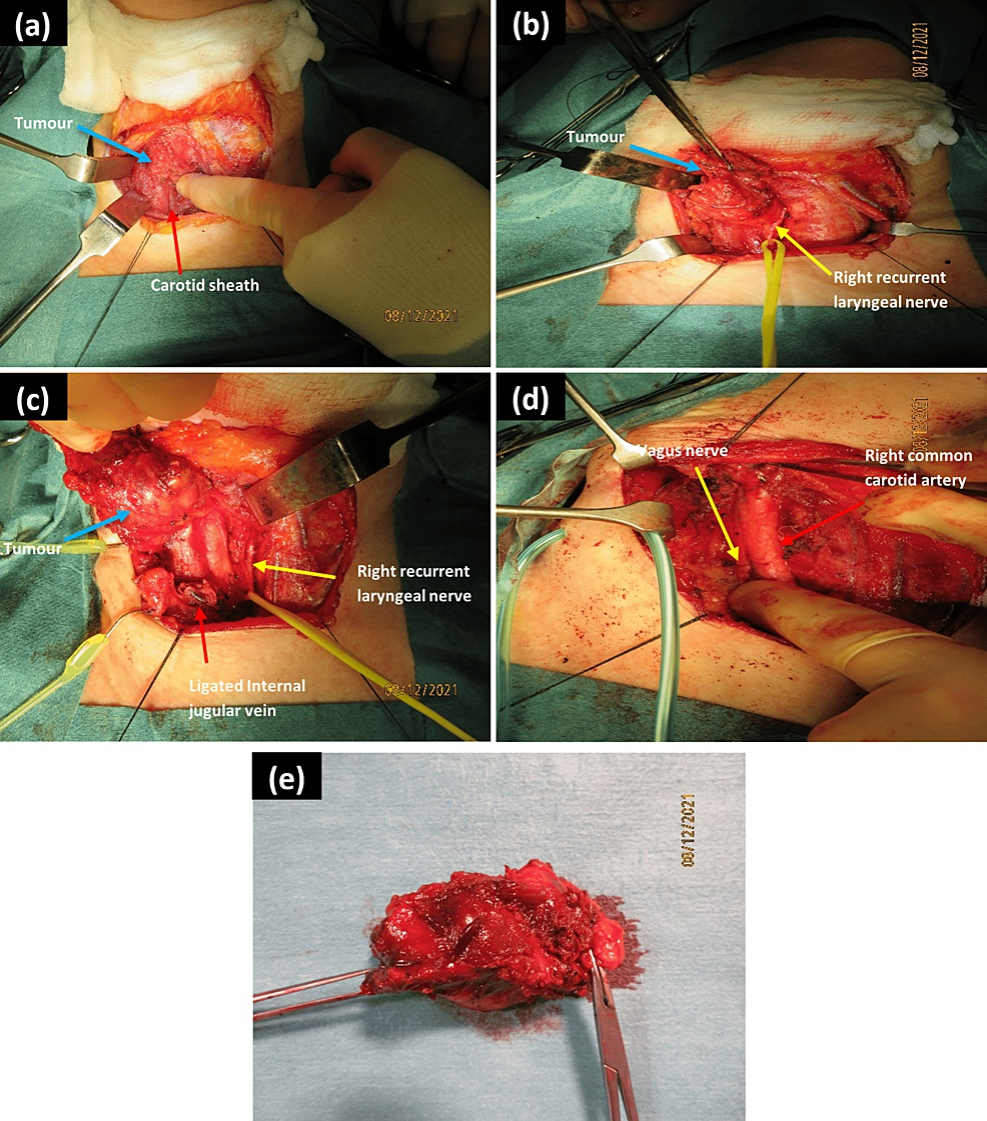
Intra-operative images (a) initial exposure of the thyroid after dividing the strap muscles and showing the proximity of the right thyroid tumour to the carotid sheath; (b) medial dissection off the trachea, with recurrent laryngeal nerve (looped by the yellow vascular loop to maximise safe tissue tension) identified and preserved; (c) superioposterior dissection, revealing the tumour being mobilised in a supero-lateral fashion after ligation of the internal jugular vein; (d) surgical field after hemithyroidectomy and neck dissection demonstrating the intact right common carotid artery and vagus nerve; (e) final specimen after excision, measuring 70 × 30 × 30 mm^3^ and weighing 27 g.

The patient was listed for a day-case right hemithyroidectomy on December 8, 2021. Although pre-operative imaging demonstrated close relation to the common carotid artery, there was no vascular surgery support for the surgical case. The procedure itself was technically challenging (Figure 4), with the right hemithyroid fixed onto the underlying tissue. Intra-operatively, it was decided to extend the procedure to include a modified ipsilateral radical neck dissection, including internal jugular vein ligation.

**Figure 5:**
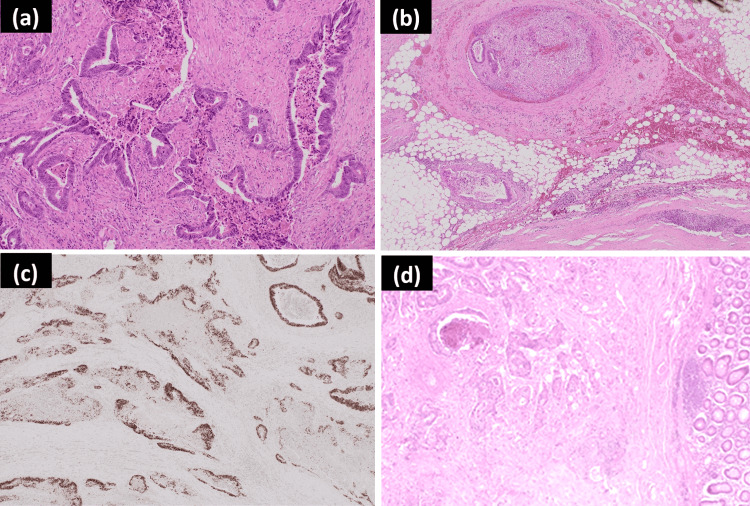
Photomicrographs of the metastatic caecal adenocarcinoma of the thyroid gland (a) Poorly differentiated caecal adenocarcinoma (haematoxylin and eosin ×10 magnification); (b) adjacent vascular invasion (haematoxylin and eosin ×4 magnification); (c) tumour positivity for CDX2 immunohistochemistry (×4 magnification); (d) comparison with the primary caecal adenocarcinoma from 2015 (haematoxylin and eosin ×4 magnification).

Histopathological analysis demonstrated that the tumour was infiltrated diffusely with a necrotic carcinoma with columnar epithelium and positivity for CDX2 and patchy positivity for CK20, confirming the diagnosis of metastatic colorectal adenocarcinoma (Figure 5). There was also direct tumour extension into lymph nodes. She completed adjuvant radiotherapy to the neck without complications. She remains well at present.

## Discussion

Colorectal cancers present a large proportion of the global cancer burden [1]. As in all cancers, metastases are associated with increased morbidity and mortality; in a Dutch population, patients with metastatic (Stage IV) colorectal cancer had a median survival of 12 months, with resection of single-site metastasis to the liver and lung providing a significant increase in median survival (p<0.001) [2]. Moreover, there is a 1-year survival rate of 44% for men and 35% for women with Stage IV colorectal cancer in England [5].

The thyroid gland, despite its rich arterial supply, is an uncommon site of clinically-evident metastasis, comprising 1.4-3% of all surgical cases, although autopsy series report a prevalence of 1.9% to 24% [6,7]. One review found an interval of 41.5 months between the diagnosis of primary colorectal cancer and thyroid metastasis, with 68% of all patients with metastases to the thyroid presenting symptomatically [4]. Symptoms included a neck lump, dysphagia, dysphonia, and cough. Moreover, 44% of cases in the review occurred in patients with pre-existing thyroid gland abnormalities, which have been purported to be due to reduced blood flow and iodine concentrations in diseased thyroid glands [4]. Finally, although equivocal, there is evidence that survival time from detection of thyroid metastasis is longer in patients undergoing surgical management, although there is no increase in overall survival [6].

Although there are case reports of colorectal cancers metastasising to the thyroid in the literature [4,8-10], this is, to the best of our knowledge, the first reported case of caecal adenocarcinoma metastasising to the thyroid. Current UK guidelines focus largely on primary thyroid cancer, with no guidance on the management of metastasis to the thyroid [11]. A diagnostic hemithyroidectomy is advised for Thy3f or Thy4 FNAC, whilst a total thyroidectomy is advised for patients with tumours greater than 4 cm in diameter, or tumours with multifocal disease, bilateral disease, extrathyroidal spread, familial disease, and evidence of metastases (from primary thyroid cancer). After a multidisciplinary team discussion, we elected to perform a hemithyroidectomy due to the oligometastatic nature of the disease, which is solely localised clinically and radiologically to the thyroid. An alternative emerging treatment paradigm in the management of this oligometastasis may be the use of stereotactic body radiotherapy (SBRT), which has shown high one- and two-year overall survival and low toxicity in the treatment of extracranial oligometastases in a UK population [12], although there is little published evidence on the treatment of metastases to the thyroid.

Operatively, as demonstrated in this case report, the surgeon needs to be adaptable based on intra-operative findings to be able to include a form of neck dissection to maximise the chance of achieving negative margins. Furthermore, if there is evidence of intra-luminal aerodigestive tract invasion macroscopically, a carefully considered decision based on patient factors, including their quality of life, would need to be made on whether to resect the macroscopic disease and reconstruct the defect based on the armamentarium of the surgeon. A pedicled pectoralis major flap used as a stented patch would be a reasonable reconstruction option. Ideally, this should be discussed with the patient during the consenting process pre-operatively in anticipation of a challenging operation.

Finally, in cases where the tumour has a close relationship to critical vascular structures such as the common carotid artery, having vascular surgery support would be optimal, even where there is no frank invasion of the common carotid artery. This is in the case of vascular injury intra-operatively or in case if there is cholangiocarcinoma (CCA) invasion and therefore for the support in the case of the need for resecting a patch of the artery and reconstruction with a patch graft. It also allows team building between head and neck and vascular for multidisciplinary team (MDT) approach to joint cases and utilises the expertise of both teams for the benefit of the patient.

## Conclusions

Our surgical case report highlights a rare metastatic site for primary caecal cancer. Although in our case, the metastasis was found incidentally, it remains important to undertake careful history-taking in patients with a background of cancer to establish any potential metastatic disease. Moreover, in challenging surgical cases such as this, pre-operative preparation should be optimised (e.g., vascular surgery support on standby). Finally, given the increased survival time for thyroid metastasis patients undergoing surgery, we would propose that, after multidisciplinary team discussion, surgery should be offered to patients with thyroid oligometastasis to attempt macroscopic disease clearance.

## References

[1] (2022). GLOBOCAN. Estimated number of new cases in 2020, worldwide, both sexes, all ages. Cancer Today. https://gco.iarc.fr/today/online-analysis-pie?v=2020&mode=cancer&mode_population=continents&population=900&populations=900&key=total&sex=0&cancer=39&type=0&statistic=5&prevalence=0&population_group=0&ages_group%5B%5D=0&ages_group%5B%5D=17&nb_items=7&group.

[2] van der Geest LG, Lam-Boer J, Koopman M, Verhoef C, Elferink MA, de Wilt JH (2015). Nationwide trends in incidence, treatment and survival of colorectal cancer patients with synchronous metastases. Clin Exp Metastasis.

[3] Riihimäki M, Hemminki A, Sundquist J, Hemminki K (2016). Patterns of metastasis in colon and rectal cancer. Sci Rep.

[4] Chung AY, Tran TB, Brumund KT, Weisman RA, Bouvet M (2012). Metastases to the thyroid: a review of the literature from the last decade. Thyroid.

[5] (2022). Office for National Statistics. Cancer survival by stage at diagnosis for England (experimental statistics): adults diagnosed 2012, 2013 and 2014 and followed up to 2015. https://www.ons.gov.uk/peoplepopulationandcommunity/healthandsocialcare/conditionsanddiseases/bulletins/cancersurvivalbystageatdiagnosisforenglandexperimentalstatistics/adultsdiagnosed20122013and2014andfollowedupto2015.

[6] Papi G, Fadda G, Corsello SM (2007). Metastases to the thyroid gland: prevalence, clinicopathological aspects and prognosis: a 10-year experience. Clin Endocrinol (Oxf).

[7] Nakhjavani MK, Gharib H, Goellner JR, van Heerden JA (1997). Metastasis to the thyroid gland: a report of 43 cases. Cancer.

[8] Amante MA, Real IO, Bermudez G (2018). Thyroid metastasis from rectal adenocarcinoma. BMJ Case Rep.

[9] Hanna WC, Ponsky TA, Trachiotis GD, Knoll SM (2006). Colon cancer metastatic to the lung and the thyroid gland. Arch Surg.

[10] Minami S, Inoue K, Irie J (2016). Metastasis of colon cancer to the thyroid and cervical lymph nodes: a case report. Surg Case Rep.

[11] Mitchell AL, Gandhi A, Scott-Coombes D, Perros P (2016). Management of thyroid cancer: United Kingdom National Multidisciplinary Guidelines. J Laryngol Otol.

[12] Chalkidou A, Macmillan T, Grzeda MT (2021). Stereotactic ablative body radiotherapy in patients with oligometastatic cancers: a prospective, registry-based, single-arm, observational, evaluation study. Lancet Oncol.

